# The Clinical Effect and Safety of Dihuang Decoction in Henoch-Schönlein Purpura Compared With Different Traditional Programs: A Network Meta-Analysis

**DOI:** 10.7759/cureus.64457

**Published:** 2024-07-13

**Authors:** Shifang Cui, Lin Liu, Fuli Zhang

**Affiliations:** 1 Traditional Chinese Internal Medicine, School of Basic Medical Sciences, Heilongjiang University of Chinese Medicine, Harbin, CHN

**Keywords:** network meta-analysis, circulation improvement, anti-inflammatory drugs, dihuang decoction, henöch-schonlein purpura

## Abstract

This systematic review aims to evaluate the therapeutic efficacy of Dihuang decoction (DD), anti-inflammatory drugs (AIDs), blood circulation improvement drugs (BCIDs), and conventional therapy (CT) in the management of Henoch-Schönlein purpura (HSP) and to establish their comparative effectiveness rankings. Using the Population, Intervention, Comparison, Outcome, Study (PICOS) design framework, we developed a detailed search strategy. The literature search included databases such as PubMed, Embase, Cochrane Library, China National Knowledge Infrastructure, Wanfang Data, Weipu Journal Data, and the Chinese Biomedical Database, covering studies published up to June 2024. We included randomized controlled trials that featured the DD as the experimental intervention, with three remaining treatments as comparators. Our analysis encompassed 63 studies with 5,435 participants, divided into 2,817 in the experimental group and 2,618 in the control group. The network meta-analysis suggested that the DD potentially surpasses AIDs, BCIDs, and CT in the management of HSP. This conclusion is supported by its superior SUCRA (Surface Under the Cumulative Ranking) scores across various measures, including the overall effective rate of medication, time to relief or disappearance of the rash, incidence of adverse reactions, time to relief or disappearance of abdominal pain, time to relief or disappearance of arthritic swelling or pain, IgA levels, and the relapse rate within six months (SUCRA scores: 100.0%, 88.3%, 79.8%, 94.4%, 99.9%, 88.3%, and 95.4%, respectively). In terms of overall effectiveness rate, the SUCRA efficacy rankings are as follows: DD > AIDs > AIDs+BCID > BCID > CT. Regarding rash relief and regression time, the SUCRA efficacy rankings are as follows: DD > CT > AIDs+BCID > AIDs. For the incidence rate of adverse reactions, the SUCRA efficacy rankings are as follows: DD > CT > AIDs > BCID > AIDs+BCID. For the relief and disappearance of abdominal pain, the SUCRA efficacy rankings are as follows: DD > CT > AIDs+BCID > AIDs. In terms of relief and disappearance of joint swelling and pain, the SUCRA efficacy rankings are as follows: DD > AIDs+BCID > AIDs. Regarding IgA changes, the SUCRA efficacy rankings are as follows: DD > CT > BCID > AIDs+BCID > AIDs. For the six-month recurrence rate, the SUCRA efficacy rankings are as follows: DD > AIDs > AIDs+BCID > CT. The DD appears to be a more effective alternative for treating HSP compared to AIDs, BCIDs, and CT. We hope that this study will provide better assistance to clinical practice.

## Introduction and background

Henoch-Schönlein purpura (HSP), also known as IgA vasculitis, is an immune complex vasculitis predominantly characterized by IgA deposition in small blood vessels. It primarily presents with skin purpura, joint pain or arthritis, acute enteritis, and glomerulonephritis [1]. A study in the field of epidemiology conducted in France revealed that the annual incidence rate of HSP in the general population is estimated to range from 3 to 26.7 cases per 100,000 among children and infants, while it is comparatively lower in adults, ranging from 0.8 to 1.8 cases per 100,000 [2]. In an epidemiological survey conducted in China, the male-to-female ratio of patients with the disease was 1.72:1, and the overall incidence showed an increasing trend year by year. Patients were affected in all four seasons, with a higher incidence in the first and fourth quarters (57.84%) and a lower incidence in the second and third quarters (42.16%) [3,4]. The complexity of the disease often correlates with specific organ involvement, with renal, pulmonary, or gastrointestinal manifestations being linked to increased morbidity and mortality [5]. The recurrent and refractory nature of HSP poses significant clinical challenges. For example, a Chinese study involving 557 patients reported that approximately 61% exhibited gastrointestinal symptoms, ranging from bleeding to severe complications like hematemesis and intestinal obstruction. Pathologically, almost all cases progress to purpuric nephritis, secondary kidney disease of HSP [6].

In Western medicine, the management of HSP predominantly focuses on supportive and symptomatic care [5]. This typically involves a combination of medications such as loratadine, hydrocortisone, cimetidine, calcium supplements, and vitamin C, along with other anti-inflammatory and circulation-enhancing drugs. These treatments aim to alleviate clinical symptoms, reduce the severity of purpura, and provide overall disease management [7]. However, other research has highlighted potential treatment-related complications, including gastrointestinal bleeding and persistent hematuria post-recovery [8,9]. In modern Western medicine research, HSP is characterized by small vessel vasculitis caused by the deposition of IgA around blood vessels and activation of neutrophils. In the pathophysiology of IgA vasculitis, immune complexes containing IgA1 with galactose-deficient type play a key role. These complexes induce inflammation of neutrophils around skin blood vessels and proliferation and inflammation of mesangium in glomeruli through the Fc α receptor (CD89). The main clinical features include round or oval-shaped palpable purpura predominantly on the lower legs, joint pain or arthritis, gastrointestinal bleeding or pain, and IgA deposition in the mesangium leading to glomerulonephritis [10]. In China, traditional Chinese medicine (TCM) has been used in the extensive treatment of HSP, and DD is a representative prescription drug. According to TCM research, the etiology and pathogenesis of HSP include exogenous heat and toxicity, deficiency of the body, dietary disorders, and stagnation of qi and blood stasis. The most common manifestation is blood stasis caused by heat and toxicity, with dense skin petechiae, or even fused into patches, with bright red or purplish red color. Dihuang decoction (DD) is a proprietary Chinese medicine composed of Radix Rehmanniae Praeparata, Radix Paeoniae Alba, and Mudan Pi [11]. Studies have reported that DD treatment of HSP can effectively improve patients' renal function, immune function, and coagulation function with remarkable clinical efficacy [12,13]. Network pharmacological studies have shown that DD can positively regulate inflammatory responses, oxidative stress, peroxidase activity, and information, making it play a crucial role in the treatment of HSP. The core targets interact with each other to influence inflammatory response, oxidative stress, and signaling, which in turn affects cell proliferation, apoptosis, metabolism, and intercellular adhesion, resulting in shorter symptom duration, enhanced resistance to infection, modulation of the immune response, and improvement of coagulation and inflammatory status [14].

The wide range of medications available for HSP treatment, along with the ambiguous efficacy rankings of these drugs, presents a complex challenge for clinicians. Although conventional meta-analyses of the efficacy of DD in HSP treatment exist [15-17], they suffer from limitations such as incomplete literature retrieval, for instance, a lack of review of references in the included articles and previous reviews in the field; and inadequate analysis of outcome measures, for example, a failure to analyze IgA levels, recurrence rates, incidence of adverse reactions, and so forth. Moreover, such analyses typically compare only two treatment modalities at a time. In contrast, network meta-analysis (NMA) offers a more comprehensive approach, integrating data from various clinical trials and interventions to assess their relative effectiveness more thoroughly. This study aims to overcome these limitations by incorporating the latest clinical research, undertaking an exhaustive review and screening of the literature, refining outcome measures, and comparing DD, anti-inflammatory drugs (AIDs), blood circulation improvement drugs (BCIDs), and conventional therapy (CT) for HSP. Based on this, the present study utilized NMA to compare the efficacy and safety of different treatment regimens for HSP, aiming to provide references and evidence for clinical therapy.

## Review

This NMA adheres to the Preferred Reporting Items for Systematic Reviews and Meta-Analyses extension for NMAs (PRISMA-NMA) guidelines [18]. The study is registered with PROSPERO (Registration number: CRD42024561751).

Search strategy

Consistent with the Participants, Intervention, Control, Outcome, Study (PICOS) design framework, we systematically searched databases including PubMed, Cochrane Library, EMBASE, China National Knowledge Infrastructure (CNKI), Weipu Journal Data, Wanfang Data, and the Chinese Biomedical Database up to June 2024. The search, conducted in both Chinese and English, combined Medical Subject Headings (MeSH) terms and free-text terms related to Henoch-Schönlein Purpura and various treatment methodologies, using Boolean operators. The English search terms for this study were "Dihuang Decoction", "Henoch-Schönlein purpura" and so on. To ensure comprehensiveness, we also reviewed references from selected articles and previous systematic reviews or overviews relevant to this field.

Eligibility

Studies were selected based on predetermined inclusion and exclusion criteria. Inclusion criteria included: (1) Patients with a confirmed diagnosis of HSP [1], without restrictions on age or gender; (2) Intervention: Experimental Group: Addition of Dihuang Decoction or Dihuang Decoction on the basis of the control group. A reasonable dose is calculated based on the patient's height, weight, and different types.; Control Group: One or more AIDs, BCIDs, and CT; (3) Study design limited to randomized controlled trials (RCTs). Exclusion criteria comprised: (1) Reviews, systematic evaluations, conference abstracts, guidelines, animal studies, case reports, theoretical articles, duplicate publications, and unrelated literature; (2) Non-randomized studies (Non-RCTs); (3) Studies with incompatible outcome measures, those not meeting inclusion criteria, or those with unextractable data; (4) Studies not involving DD in the experimental group.

Data extraction

Two independent researchers conducted the data extraction process, strictly adhering to the inclusion and exclusion criteria. Cross-verification was performed to ensure accuracy. Information extracted included: (1) References (first author, publication year); (2) Population details (sample size, gender distribution, age range); (3) Intervention specifics (DD, AIDs, BCID, CT); (4) Outcome measures (overall effective rate of medication, time to relief or disappearance of the rash, incidence of adverse reactions, time to relief or disappearance of abdominal pain, time to relief or disappearance of arthritic swelling or pain, IgA levels, relapse rate within six months).

For continuous outcomes (time to relief or disappearance of the rash, time to relief or disappearance of abdominal pain, time to relief or disappearance of arthritic swelling or pain, IgA levels), mean changes and standard deviations from baseline were extracted or calculated for both treatment and control groups. For dichotomous outcomes (overall effective rate of medication, incidence of adverse reactions, relapse rate within six months), the total number of cases and events for each treatment was determined.

Quality assessment

The evaluation of the included studies was meticulously conducted across six distinct domains, utilizing the Cochrane Risk of Bias Tool (RoB 2.0) [19]. This comprehensive assessment encompassed six biases: randomization process, deviations from intended interventions, missing outcome data, measurement of the outcome, selection of the reported result, and overall bias. Each study was independently assessed by two researchers, categorizing each domain as "low risk," "high risk," or "some concerns." Discrepancies were resolved through discussion or third-party consultation. The assessment results were illustrated in a risk of bias graph.

Statistical analysis

The NMA was conducted using STATA version 14.0. A network plot provided a succinct summary of the evidence for each intervention. Odds ratios (ORs) for dichotomous variables and mean differences (MD) for continuous variables were used, with 95% confidence interval (CI) calculated and reported. If the ORs do not include 1, or if the mean differences do not include 0, then a P-value of less than 0.05 is considered statistically significant. Global consistency and node-splitting methods were applied to check for inconsistency (P<0.05 indicating inconsistency). A comparison-adjusted funnel plot was drawn to assess small study effects. The Surface Under the Cumulative Ranking Curve (SUCRA) was used for intervention ranking, with higher SUCRA values indicating greater efficacy or probability of ranking. Indirect pairwise comparisons among the four interventions were summarized in a league table.

Results

Characteristics of Included Studies

**Figure 1:**
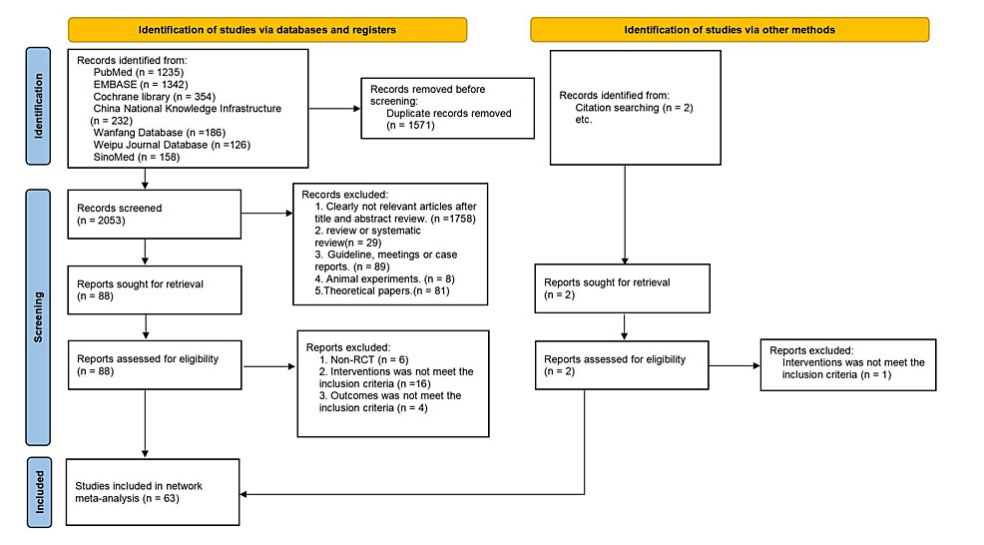
Flowchart. RCT: Randomized controlled trial

Our NMA evaluated 63 studies [9-11,20-79], comprising a total of 5,435 participants (Figure 1). All these studies, conducted in China and published up to June 2024, were RCTs. The sample sizes ranged from 30 to 246 patients diagnosed with HSP. The detailed characteristics of these studies are presented in Table 1.

**Table 1:** Characteristics of the included studies. DD: Dihuang Decoction; AIDs: Anti-inflammatory Drugs; BCID: Blood Circulation Improvement Drug; CT: Conventional Therapy.

Study ID	Experimental group				Control group				Outcomes
	Sample size	Sex(M\F)	Age(years)	Intervention	Sample size	Sex(M\F)	Age(years)	Interventions	
García-Porrúa et al. 2017 [9]	57	30/27	7.13±2.19	DD	57	29/28	6.94±2.41	AIDs+BCID	Overall effective rate of medication; time to relief or disappearance of the rash; incidence of adverse reactions; time to relief or disappearance of abdominal pain; time to relief or disappearance of arthritic swelling or pain
Pillebout 2021 [10]	40	-	-	DD	40	-	-	AIDs	Overall effective rate of medication; time to relief or disappearance of the rash; incidence of adverse reactions; time to relief or disappearance of abdominal pain; time to relief or disappearance of arthritic swelling or pain
Wenliang 2018 [11]	50	35/15	10.6±1.0	DD	50	30/20	10.2±1.2	AIDs	Overall effective rate of medication
Lifang 2003 [20]	38	23/15	8.3	DD	30	18/12	8.2	AIDs+BCID	Overall effective rate of medication
Yuhong 2019 [21]	55	41/14	6.79±2.55	DD	55	40/15	6.75±2.53	AIDs+BCID	Overall effective rate of medication; time to relief or disappearance of the rash; time to relief or disappearance of abdominal pain; time to relief or disappearance of arthritic swelling or pain
Huixing 2005 [22]	35	-	-	DD	33	-	-	AIDs+BCID	Overall effective rate of medication
Jun 2012 [23]	45	26/19	11.53±4.55	DD	45	23/22	11.62±4.83	AIDs+BCID	Overall effective rate of medication; relapse rate within six months
Yan et al. 2017 [24]	20	9/11	12.5±5.3	DD	20	12/8	11.6±6.7	AIDs+BCID	Overall effective rate of medication
Shaoping 2013 [25]	23	-	-	DD	22	-	-	AIDs+BCID	Overall effective rate of medication; time to relief or disappearance of the rash; time to relief or disappearance of arthritic swelling or pain
Jinghua 2010 [26]	32	20/12	7.65	DD	32	19/l3	7.25	AIDs+BCID	Overall effective rate of medication
Changjiang et al. 2017 [27]	81	49/32	6.71±2.52	DD	27	16/11	7.01±2.73	AIDs+BCID	Overall effective rate of medication; time to relief or disappearance of the rash; incidence of adverse reactions; time to relief or disappearance of abdominal pain; time to relief or disappearance of arthritic swelling or pain
Haiming 2017 [28]	42	25/17	6.5±1.4	DD	42	24/18	6.9±1.2	AIDs+BCID	Overall effective rate of medication; IgA levels
Xiaowen et al. 2009 [29]	44	-	-	DD	48	-	-	AIDs+BCID	Overall effective rate of medication; relapse rate within six months
Xuhe et al. 2021 [30]	60	36/24	9.67±1.35	DD	30	18/12	9.85±1.37	CT	incidence of adverse reactions; relapse rate within six months
Suyun et al. 2018 [31]	76	40/36	5.40±3.20	DD	76	38/38	5.20±3.40	AIDs+BCID	Overall effective rate of medication; time to relief or disappearance of the rash; time to relief or disappearance of abdominal pain; time to relief or disappearance of arthritic swelling or pain
Yan et al. 2012 [32]	30	20/10	8.5	DD	30	21/9	8	AIDs+BCID	Overall effective rate of medication; time to relief or disappearance of the rash; time to relief or disappearance of abdominal pain; time to relief or disappearance of arthritic swelling or pain; relapse rate within six months
Qingfeng 2012 [33]	46	26/20	23.3±16.9	DD	46	25/21	19.8±15.3	AIDs+BCID	Overall effective rate of medication; time to relief or disappearance of abdominal pain; time to relief or disappearance of arthritic swelling or pain; relapse rate within six months
Tusheng 2010 [34]	35	20/15	7	DD	35	21/14	7	AIDs+BCID	Overall effective rate of medication
Linlin et al. 2016 [35]	87	42/45	8.35±2.76	DD	87	46/41	8.42±2.57	AIDs+BCID	Incidence of adverse reactions; IgA levels; relapse rate within six months
Haoliang 2014 [36]	65	30/35	14.8	DD	63	29/34	15.3	AIDs+BCID	Overall effective rate of medication
Li et al. 2005 [37]	90	53/37	9.56±1.17	DD	90	59/31	9.69±2.26	AIDs+BCID	Overall effective rate of medication; relapse rate within six months
Dongyan 2021 [38]	23	15/8	8.7±1.3	DD	22	13/9	8.6±1.2	AIDs+BCID	Overall effective rate of medication; time to relief or disappearance of the rash; time to relief or disappearance of arthritic swelling or pain
Haili 2014 [39]	40	27/13	7.81±2.87	DD	40	29/11	7.60±3.14	AIDs+BCID	Overall effective rate of medication; time to relief or disappearance of the rash; time to relief or disappearance of arthritic swelling or pain
Dali et al. 2016 [40]	45	21/24	7.7±2.8	DD	45	22/23	7.5±3.1	AIDs+BCID	Overall effective rate of medication; time to relief or disappearance of the rash; incidence of adverse reactions; time to relief or disappearance of abdominal pain; time to relief or disappearance of arthritic swelling or pain
Bohua et al. 2013 [41]	20	10/10	-	DD	20	11/9	-	AIDs+BCID	Overall effective rate of medication
Xinchun et al. 2014 [42]	58	36/22	-	DD	52	30/22	-	BCID	Overall effective rate of medication
Shuting 2019 [43]	30	15/15	-	DD	30	16/14	-	AIDs	Overall effective rate of medication; incidence of adverse reactions
Yang 2017 [44]	55	40/15	5.47±3.11	DD	55	35/20	5.68±3.09	BCID	Overall effective rate of medication
Lingyan et al. 2015 [45]	22	11/11	8.31±1.45	DD	23	12/11	8.13±1.32	AIDs+BCID	Overall effective rate of medication
Shijing 2018 [46]	61	33/28	5.8±1.1	DD	61	34/27	6.0±1.2	AIDs	Overall effective rate of medication
Hongzhi et al. 2021 [47]	20	15/5	3.94±1.06	DD	20	12/8	4.17±1.03	CT	Overall effective rate of medication; incidence of adverse reactions; IgA levels
Huimin 2021 [48]	30	17/13	-	DD	30	17/13	-	AIDs+BCID	Overall effective rate of medication
Xiaoning 2020 [49]	110	62/48	7.7±3.5	DD	110	60/50	7.6±3.3	AIDs+BCID	Overall effective rate of medication; time to relief or disappearance of the rash; incidence of adverse reactions time to relief or disappearance of arthritic swelling or pain; IgA levels
Yanli et al. 2019 [50]	53	33/20	10.64±2.12	DD	53	34/19	10.37±2.41	BCID	Overall effective rate of medication; incidence of adverse reactions; IgA levels
Na 2023 [51]	49	26/23	7.3±2.2	DD	49	27/22	7.6±2.3	AIDs	Overall effective rate of medication; incidence of adverse reactions; IgA levels; relapse rate within six months
Chundong et al. 2022 [52]	45	23/22	32.96±8.95	DD	45	25/20	36.51±10.87	AIDs+BCID	Overall effective rate of medication
Jianhui 2014 [53]	40	27/13	-	DD	40	29/11	-	AIDs+BCID	Overall effective rate of medication; time to relief or disappearance of the rash; time to relief or disappearance of arthritic swelling or pain
Yannan et al. 2021 [54]	70	41/29	9.81±1.75	DD	70	39/31	9.73±1.28	AIDs+BCID	Overall effective rate of medication; IgA levels
Guo 2015 [55]	30	17/13	7.83±2.53	DD	30	11/19	7.27±2.43	AIDs+BCID	Overall effective rate of medication; incidence of adverse reactions
Huang 2022 [56]	25	13/12	8.67±1.75	DD	25	14/11	8.96±1.84	AIDs+BCID	Overall effective rate of medication; IgA levels
Liu 2018 [57]	30	18/12	26.66±7.07	DD	30	16/14	25.93±5.77	AIDs+BCID	Overall effective rate of medication; incidence of adverse reactions
Xiaoya et al. 2018 [58]	40	22/18	7.3±1.6	DD	40	19/21	7.7±2.6	AIDs+BCID	Overall effective rate of medication
Junliang et al. 2019 [59]	35	20/15	7.11±2.52	DD	35	19/16	7.34±2.62	CT	Overall effective rate of medication; time to relief or disappearance of the rash; time to relief or disappearance of abdominal pain
Xiping 2011 [60]	30	17/13	-	DD	30	16/14	-	AIDs+BCID	Overall effective rate of medication; time to relief or disappearance of the rash; time to relief or disappearance of arthritic swelling or pain
Dongye 2011 [61]	50	-	-	DD	40	-	-	AIDs+BCID	Overall effective rate of medication
Qin 2007 [62]	35	21/14	-	DD	35	23/12	-	AIDs+BCID	Overall effective rate of medication
Huang 2017 [63]	30	20/10	7.60±3.14	DD	30	22/8	7.81±2.87	CT	Overall effective rate of medication
Xiangyu 2020 [64]	40	18/22	10.98±2.41	DD	40	21/19	11.01±2.36	AIDs+BCID	Overall effective rate of medication; time to relief or disappearance of the rash; time to relief or disappearance of abdominal pain; time to relief or disappearance of arthritic swelling or pain
Longfei 2018 [65]	30	18/12	5.7±0.9	DD	30	16/14	5.3±0.6	AIDs+BCID	Overall effective rate of medication; time to relief or disappearance of the rash; time to relief or disappearance of abdominal pain
Xiaohua 2016 [66]	44	28/16	9.76±2.03	DD	44	26/18	9.81±2.12	AIDs+BCID	Overall effective rate of medication; time to relief or disappearance of the rash; time to relief or disappearance of arthritic swelling or pain
Xuhong et al. 1995 [67]	32	-	-	DD	20	-	-	AIDs+BCID	Overall effective rate of medication
Xiaolin 2006 [68]	30	19/11	-	DD	25	17/8	-	AIDs+BCID	Overall effective rate of medication
Xiaolin 2011 [69]	15	9/6	8.5 ± 1.1	DD	15	8/7	8.5 ± 1.2	CT	Overall effective rate of medication
Guimu 2019 [70]	60	40/20	-	DD	30	18/12	-	AIDs	Overall effective rate of medication
Zhou 2008 [71]	20	-	-	DD	20	-	-	AIDs+BCID	Overall effective rate of medication
Shenglin 2013 [72]	36	20/16	6.17±1.36	DD	36	19/17	6.23±1.24	AIDs+BCID	Overall effective rate of medication; time to relief or disappearance of the rash
Ping 2018 [73]	57	-	-	DD	43	-	-	AIDs+BCID	Overall effective rate of medication
Xiuli 1998 [74]	126	-	-	DD	120	-	-	AIDs+BCID	Time to relief or disappearance of the rash; time to relief or disappearance of arthritic swelling or pain
Fenghai 2011 [75]	50	22/28	21	DD	30	14/16	20	AIDs	Overall effective rate of medication
Keqin 2005 [76]	43	20/23	7.7±2.83	DD	40	19/21	8.00±2.50	AIDs+BCID	Overall effective rate of medication
Chuanjia 2012 [77]	36	19/17	28.3±4.2	DD	36	16/20	32.5±5.9	AIDs+BCID	Overall effective rate of medication
Maojun 2010 [78]	31	16/15	8.77±1.12	DD	31	18/13	8.52±1.06	AIDs+BCID	Time to relief or disappearance of the rash; time to relief or disappearance of abdominal pain; time to relief or disappearance of arthritic swelling or pain; IgA levels
Bingxia 2021 [79]	40	-	-	DD	40	-	-	AIDs+BCID	Overall effective rate of medication

Methodological Quality

**Figure 2:**
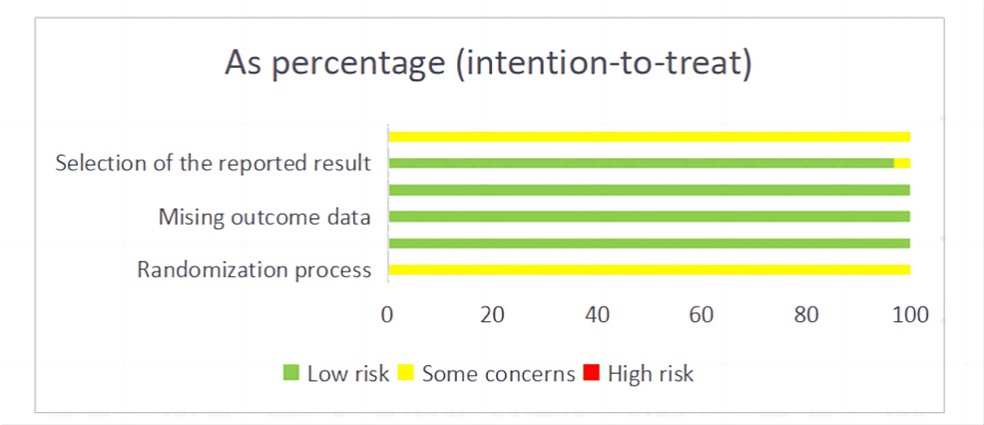
Assessment figure of risk of bias.

The 63 included studies described the generation of random sequences, but none explicitly detailed the plans for concealing the allocation in randomized controlled trials, indicating a potential risk of bias. Two studies were identified as having a potential risk due to incomplete reporting of pre-specified outcome indicators. However, all studies were deemed to have a low risk of bias regarding deviations from intended interventions, missing outcome data, and the measurement of outcomes. The quality and methodological assessments of the included studies are visually summarized in Figure 2, indicating a generally low risk of bias across the literature.

Network Diagram

**Figure 3:**
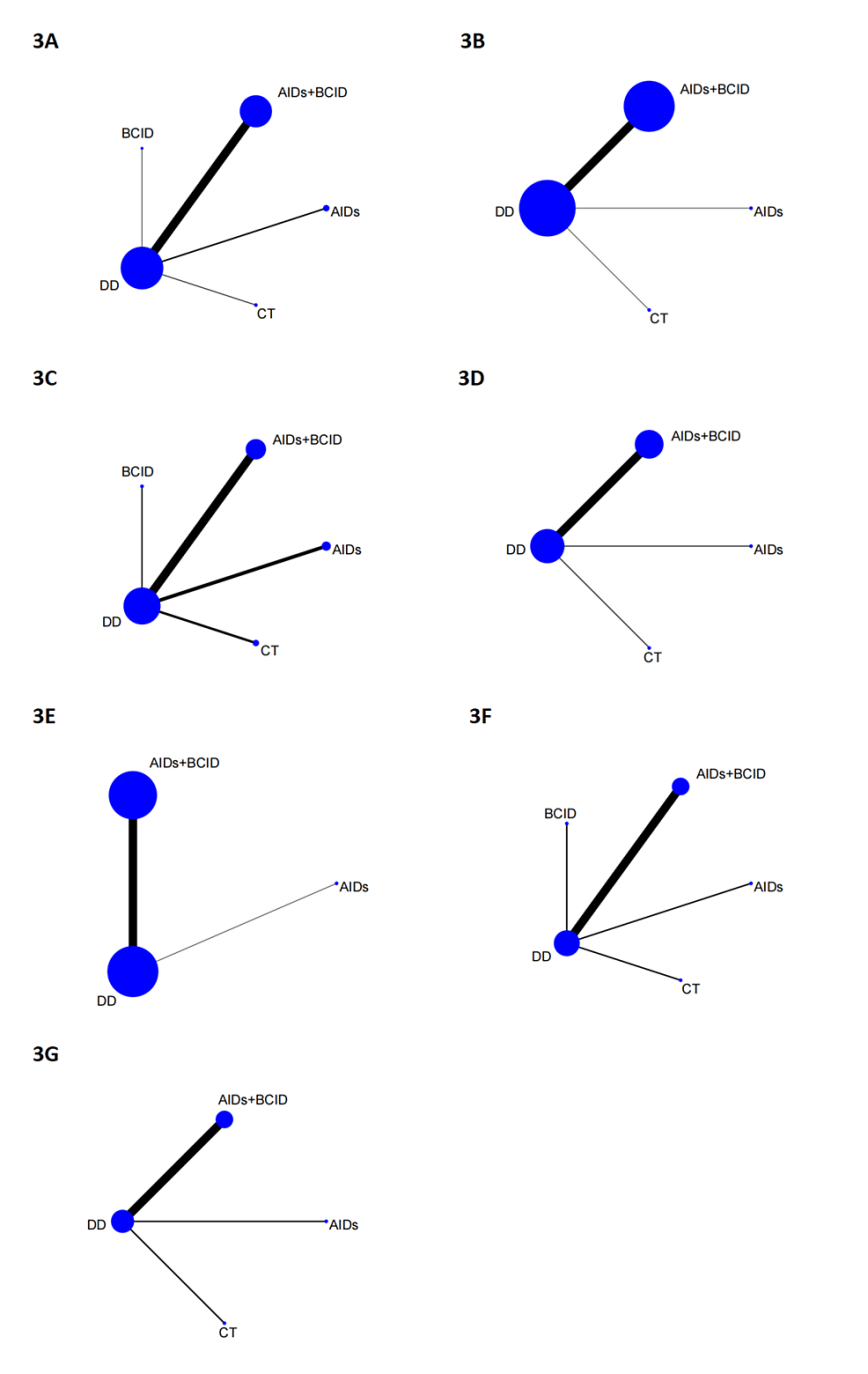
Network diagram. (A) Overall effective rate of medication. (B) Time to relief or disappearance of the rash. (C) Incidence of adverse reactions. (D) Time to relief or disappearance of abdominal pain. (E) Time to relief or disappearance of arthritic swelling or pain. (F) IgA levels. (G) Relapse rate within six months. DD: Dihuang Decoction; AIDs: Anti-inflammatory Drugs; BCID: Blood Circulation Improvement Drug; CT: Conventional Therapy.

Figure 3 illustrates the network geometry, showing comparisons among different intervention measures. The size of each circle reflects the number of participants in the studies associated with that intervention, and the thickness of the lines indicates the number of studies comparing these interventions.

Comparative Efficacy of Different Interventions

**Figure 4:**
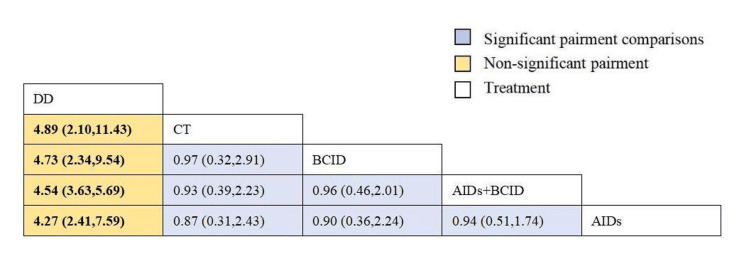
League table of the overall effective rate of medication. DD: Dihuang Decoction; AIDs: Anti-inflammatory Drugs; BCID: Blood Circulation Improvement Drug; CT: Conventional Therapy.

**Figure 5:**
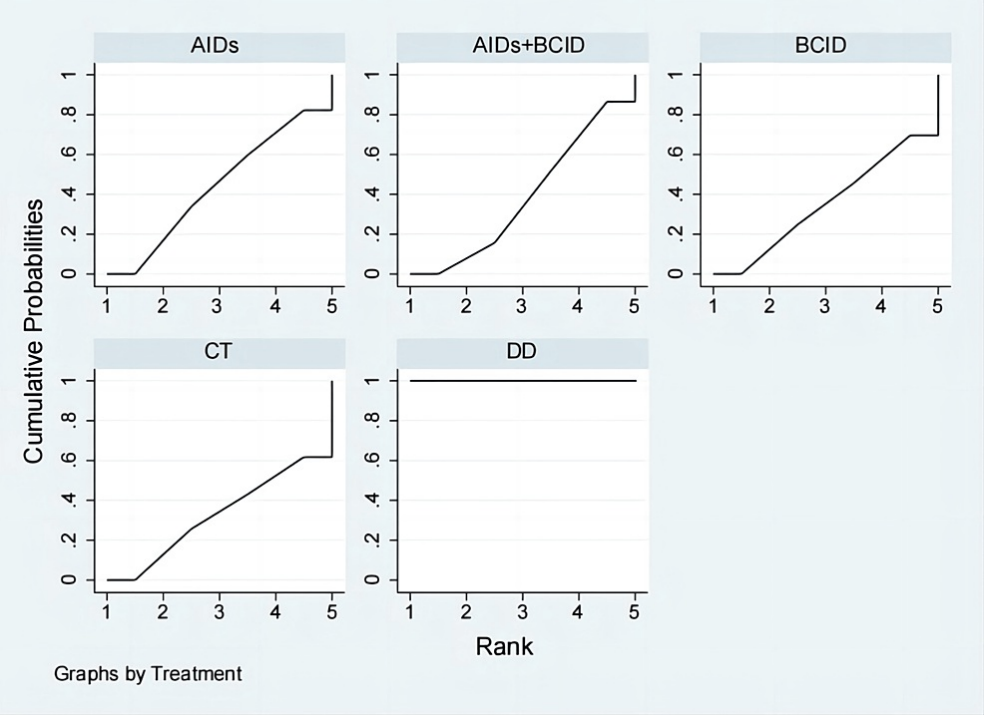
Cumulative probability line chart of the overall effective rate of medication. DD: Dihuang Decoction; AIDs: Anti-inflammatory Drugs; BCID: Blood Circulation Improvement Drug; CT: Conventional Therapy.

Fifty-nine studies with 4,861 participants assessed the overall effective rate of medication [9-11,20-29,31-34,36-73,75-77,79]. Global consistency tests yielded a significant result (P < 0.05). In point-to-point comparisons, no significant difference was found (P > 0.05) (Table 2). A consistency model was used for comparison. The DD proved to be more effective than anti-inflammatory drugs, circulation-improving drugs, a combination of anti-inflammatory and circulation-improving drugs, and conventional treatment in terms of overall medication effectiveness (OR = 4.27, 95% CI: 2.41,7.59; OR = 4.54, 95% CI: 3.63,5.69; OR = 4.73, 95% CI: 2.34,9.54; OR = 4.89, 95% CI: 2.10,11.43), with statistically significant differences. No significant differences were observed in the effects of other interventions (Figure 4). According to SUCRA results, the DD was the most effective (SUCRA = 100.0%) (Figure 5).

**Table 2:** Nodal splitting data sheet of the overall effective rate of medication. DD: Dihuang Decoction; AIDs: Anti-inflammatory Drugs; BCID: Blood Circulation Improvement Drug; CT: Conventional Therapy.

Side	Direct		Indirect		Difference			tau
	Coef.	Std. Err.	Coef.	Std.Err.	Coef.	Std.Err.	P>z	
AIDs DD *	1.506232	0.4459187	0.9562509	15.60855	0.5499815	15.61492	0.972	2.13E-08
AIDs+BCID DD *	1.580589	0.1448377	2.666697	32.8588	-1.086107	32.85944	0.974	9.02E-10
BCID DD *	1.655511	0.4688494	2.654934	126.9674	-0.9994236	126.9691	0.994	6.71E-09
CT DD *	1.185624	0.735408	2.79064	163.4479	-1.605016	163.4507	0.992	2.58E-09

**Figure 6:**
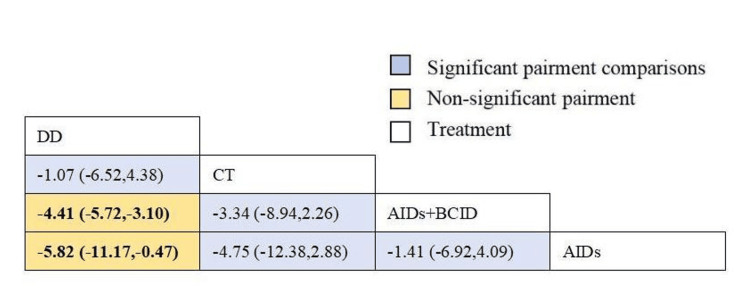
League table of time to relief or disappearance of the rash. DD: Dihuang Decoction; AIDs: Anti-inflammatory Drugs; BCID: Blood Circulation Improvement Drug; CT: Conventional Therapy.

**Figure 7:**
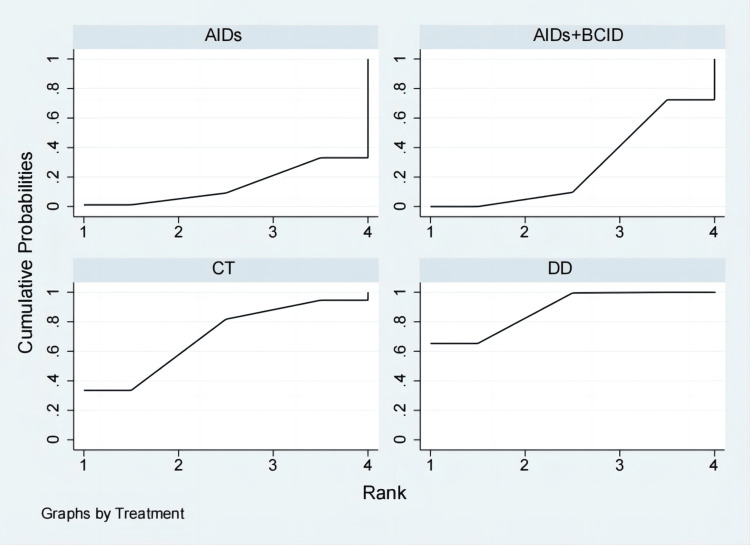
Cumulative probability line chart of time to relief or disappearance of the rash. DD: Dihuang Decoction; AIDs: Anti-inflammatory Drugs; BCID: Blood Circulation Improvement Drug; CT: Conventional Therapy.

Twenty studies with 1,920 participants assessed the time to relief or disappearance of the rash [9,10,21,25,27,31,32,38-40,49,53,59,60,64-66,72,74,78]. Global consistency tests showed no significant difference (P > 0.05), and a consistency model was used for comparison. The DD proved to be more effective than anti-inflammatory drugs and a combination of anti-inflammatory and circulation-improving drugs in terms of rash relief and disappearance times (MD = -5.82, 95% CI: -11.17,-0.47; MD = -4.41, 95% CI: -5.72,-3.10), with statistically significant differences. No significant differences were observed in other interventions (Figure 6). According to SUCRA results, the DD was the most effective (SUCRA = 88.3%) (Figure 7).

**Figure 8:**
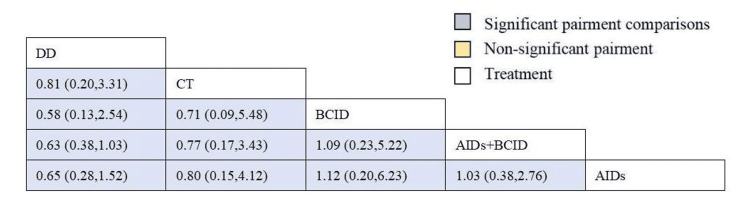
League table of incidence of adverse reactions. DD: Dihuang Decoction; AIDs: Anti-inflammatory Drugs; BCID: Blood Circulation Improvement Drug; CT: Conventional Therapy.

**Figure 9:**
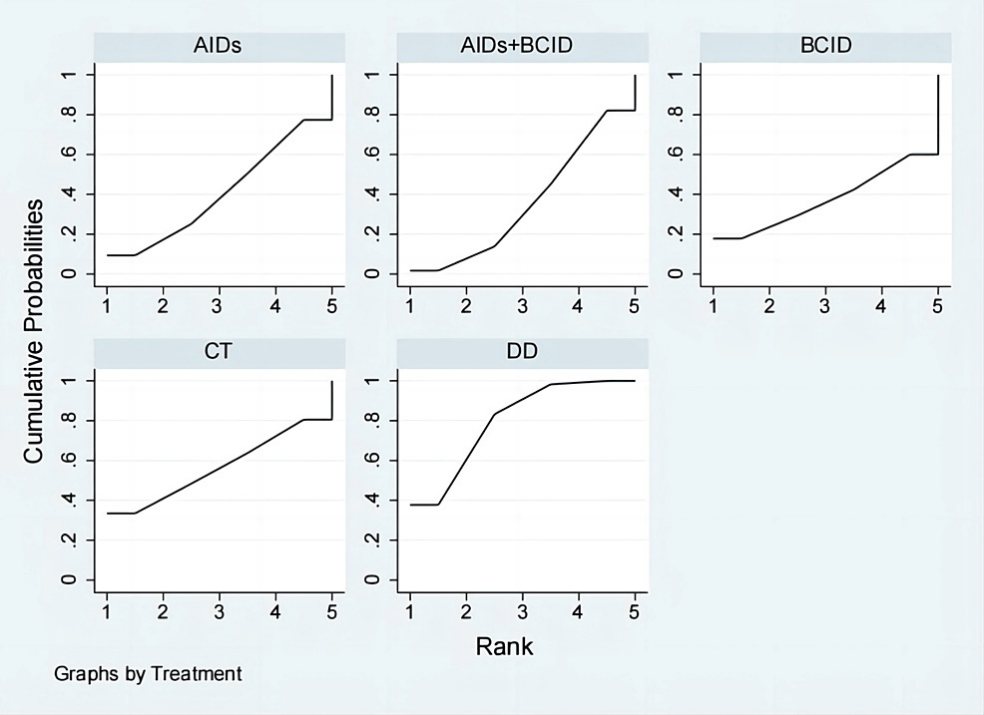
Cumulative probability line chart of incidence of adverse reactions. DD: Dihuang Decoction; AIDs: Anti-inflammatory Drugs; BCID: Blood Circulation Improvement Drug; CT: Conventional Therapy.

Thirteen studies involving 1,300 participants evaluated the incidence of adverse reactions [9,10,27,30,35,40,43,47,49-51,55,57]. Global consistency tests showed no significant difference (P > 0.05), and a consistency model was used for comparison. No significant differences were found in the incidence of adverse reactions among the interventions (Figure 8). According to SUCRA results, the DD was the most effective (SUCRA = 79.8%) (Figure 9).

**Figure 10:**
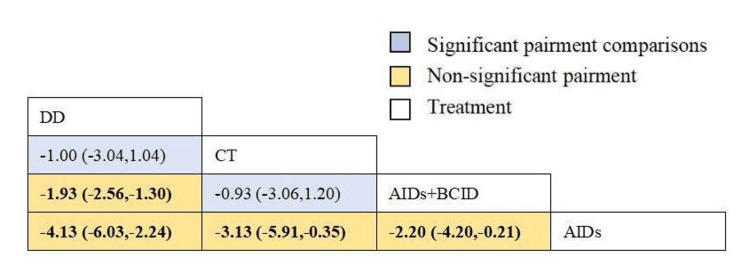
League table of time to relief or disappearance of abdominal pain. DD: Dihuang Decoction; AIDs: Anti-inflammatory Drugs; BCID: Blood Circulation Improvement Drug; CT: Conventional Therapy.

**Figure 11:**
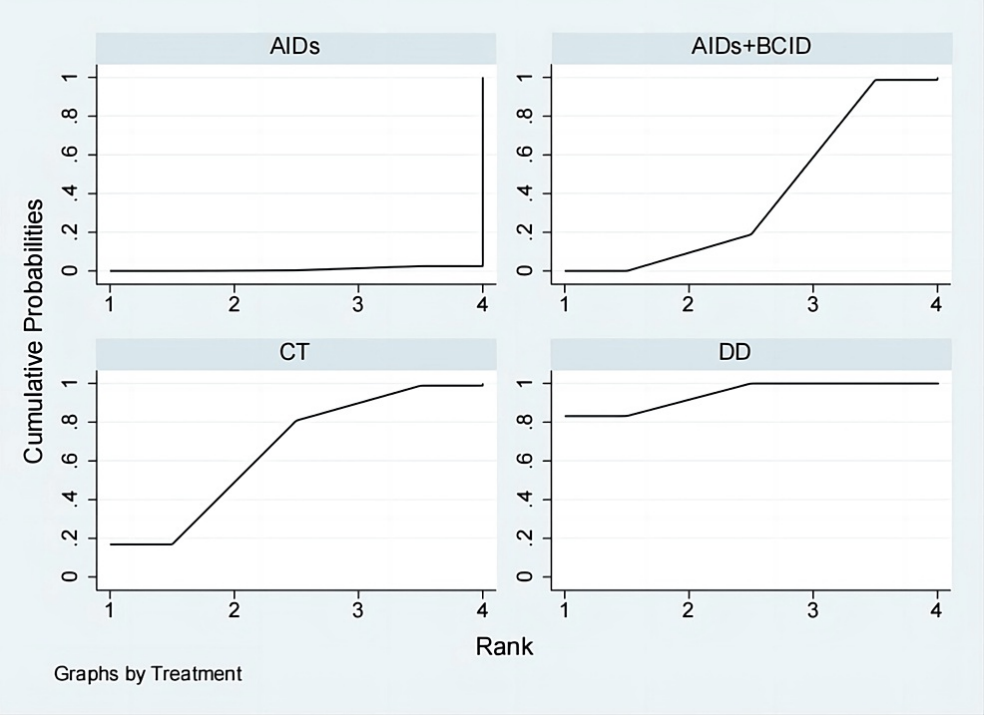
Cumulative probability line chart of time to relief or disappearance of abdominal pain. DD: Dihuang Decoction; AIDs: Anti-inflammatory Drugs; BCID: Blood Circulation Improvement Drug; CT: Conventional Therapy.

Twelve studies with 1,076 participants assessed the time to relief or disappearance of abdominal pain [9,10,21,27,31-33,40,59,64,65,78]. Global consistency tests showed no significant difference (P > 0.05), and a consistency model was used for comparison. The DD proved to be more effective than anti-inflammatory drugs and a combination of AIDs and BCIDs in terms of abdominal pain relief and disappearance times (MD = -4.13, 95% CI: -6.03,-2.24; MD = -1.93, 95% CI: -2.56,-1.30). The conventional treatment was more effective than AIDs (MD = -3.13, 95% CI: -5.91,-0.35), and the combination of AIDs and BCIDs was more effective than AIDs alone (MD = -2.20, 95% CI: -4.20,-0.21), with statistically significant differences. No significant differences were found in other interventions (Figure 10). According to SUCRA results, the DD was the most effective (SUCRA = 94.4%) (Figure 11).

**Figure 12:**
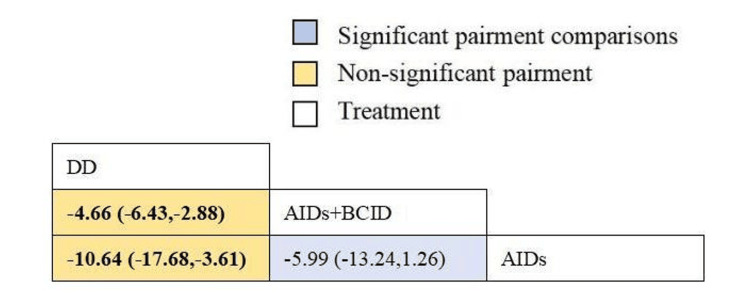
League table of time to relief or disappearance of arthritic swelling or pain. DD: Dihuang Decoction; AIDs: Anti-inflammatory Drugs; BCID: Blood Circulation Improvement Drug; CT: Conventional Therapy.

**Figure 13:**
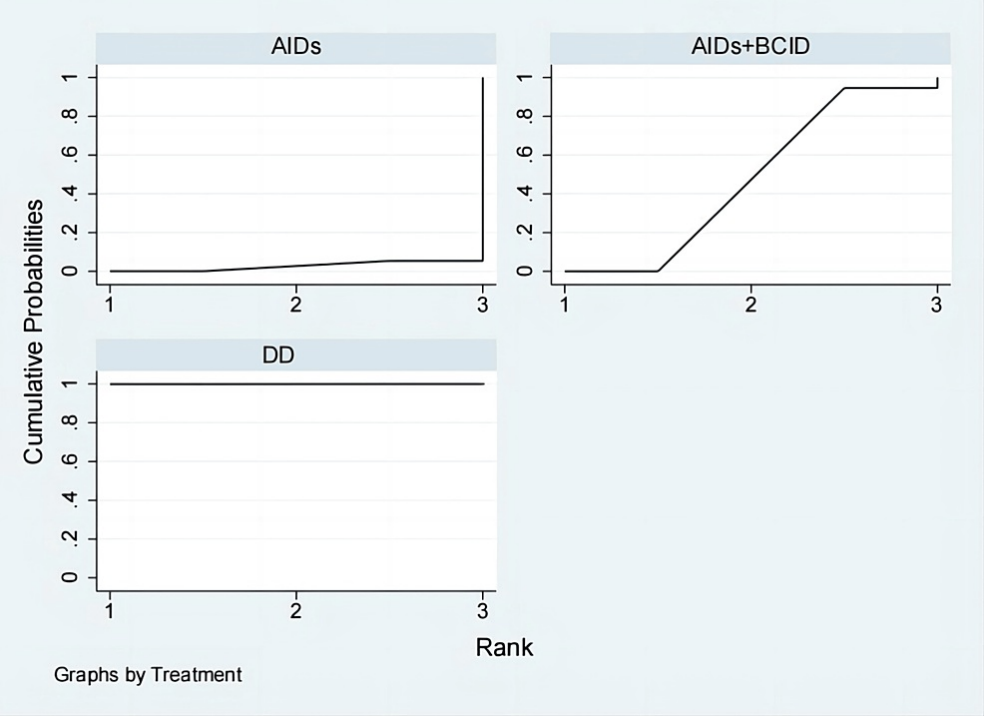
Cumulative probability line chart of time to relief or disappearance of arthritic swelling or pain. DD: Dihuang Decoction; AIDs: Anti-inflammatory Drugs; BCID: Blood Circulation Improvement Drug; CT: Conventional Therapy.

Eighteen studies involving 1,810 participants assessed the time to relief or disappearance of arthritic swelling or pain [9,10,21,25,27,31-33,38-40,49,52,60,64,66,74,78]. Global consistency tests indicated a significant result (P < 0.05). In point-to-point comparisons, no significant difference was found (P > 0.05) (Table 3). A consistency model was used for comparison. The cornus DD proved to be more effective than a combination of AIDs and BCIDs, and AIDs in terms of joint swelling and pain relief and disappearance times (MD = -4.66, 95% CI: -6.43,-2.88; MD = -10.64, 95% CI: -17.68,-3.61), with statistically significant differences. No significant differences were found in other interventions (Figure 12). According to SUCRA results, the DD was the most effective (SUCRA = 99.9%) (Figure 13).

**Table 3:** Nodal splitting data sheet of time to relief or disappearance of arthritic swelling or pain. DD: Dihuang Decoction; AIDs: Anti-inflammatory Drugs; BCID: Blood Circulation Improvement Drug; CT: Conventional Therapy.

Side	Direct		Indirect		Difference			tau
	Coef.	Std. Err.	Coef.	Std. Err.	Coef.	Std. Err.	P>z	
AIDs DD *	-11.05	2.769971	-1.689612	17.11215	-9.360388	17.33489	0.589	2.726375
AIDs+BCID DD *	-3.485116	0.7495198	-22.20589	34.66196	18.72078	34.66989	0.589	2.726375

**Figure 14:**
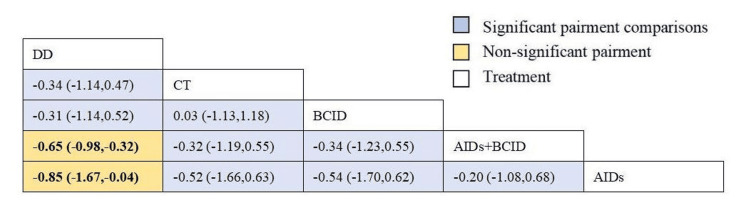
League table of IgA levels. DD: Dihuang Decoction; AIDs: Anti-inflammatory Drugs; BCID: Blood Circulation Improvement Drug; CT: Conventional Therapy.

**Figure 15:**
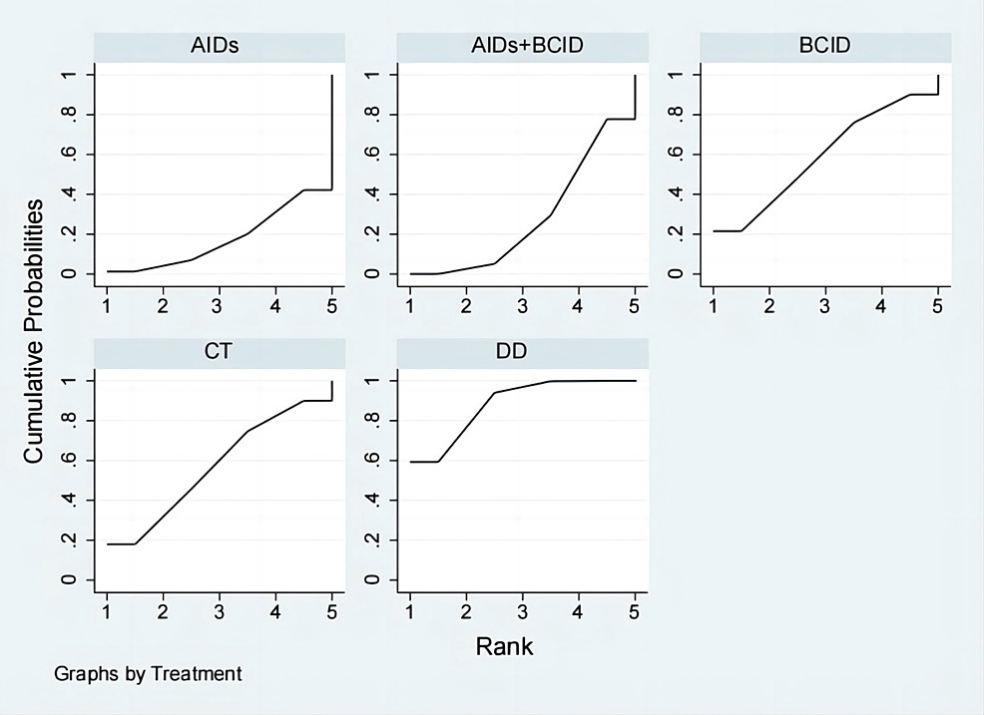
Cumulative probability line chart of IgA levels. DD: Dihuang Decoction; AIDs: Anti-inflammatory Drugs; BCID: Blood Circulation Improvement Drug; CT: Conventional Therapy.

Nine studies with 974 participants assessed IgA levels [28,35,47,49-51,54,56,78]. Global consistency tests showed no significant difference (P > 0.05), and a consistency model was used for comparison. The DD proved to be more effective than AIDs and a combination of AIDs and BCIDs in terms of IgA levels (MD = -0.85, 95% CI: -1.67,-0.04; MD = -0.65, 95% CI: -0.98,-0.32), with statistically significant differences. No significant differences were found in other interventions (Figure 14). According to SUCRA results, the DD was the most effective (SUCRA = 88.3%) (Figure 15).

**Figure 16:**
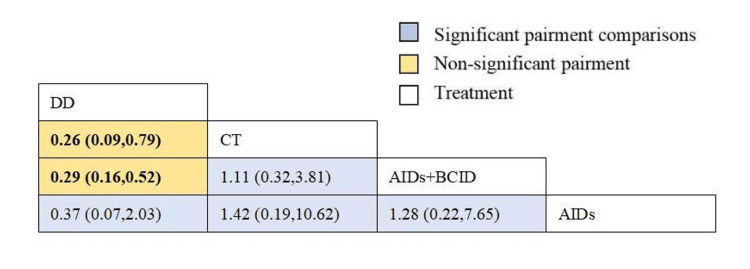
League table of relapse rate within six months. DD: Dihuang Decoction; AIDs: Anti-inflammatory Drugs; BCID: Blood Circulation Improvement Drug; CT: Conventional Therapy.

**Figure 17:**
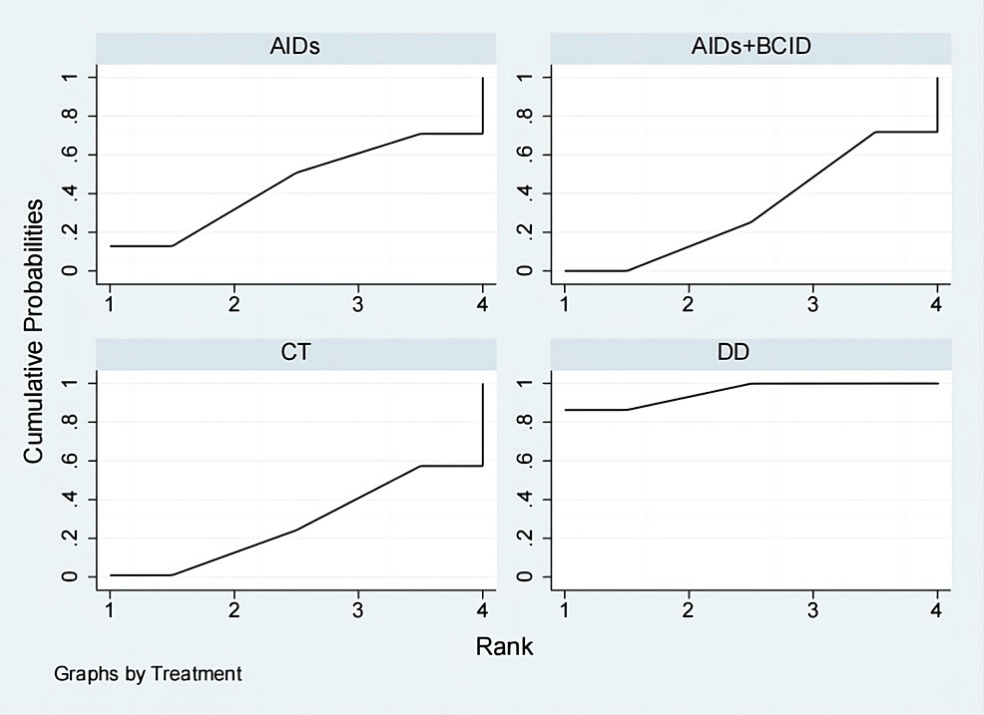
Cumulative probability line chart of relapse rate within six months. DD: Dihuang Decoction; AIDs: Anti-inflammatory Drugs; BCID: Blood Circulation Improvement Drug; CT: Conventional Therapy.

Eight studies involving 860 participants assessed the recurrence rate within six months [23,29,30,32,33,35,37,51]. Global consistency tests indicated a significant result (P < 0.05). In point-to-point comparisons, no significant difference was found (P > 0.05) (Table 4). The DD proved to be more effective than both a combination of AIDs and BCIDs and the conventional treatment in terms of the six-month recurrence rate (OR = 0.29, 95% CI: 0.16,0.52; OR = 0.26, 95% CI: 0.09,0.79), with statistically significant differences. No significant differences were found in other interventions (Figure 16). According to SUCRA results, the cornus DD was the most effective (SUCRA = 95.4%) (Figure 17).

**Table 4:** Nodal splitting data sheet of relapse rate within six months. DD: Dihuang Decoction; AIDs: Anti-inflammatory Drugs; BCID: Blood Circulation Improvement Drug; CT: Conventional Therapy.

Side	Direct		Indirect		Difference			tau
	Coef.	Std. Err.	Coef.	Std. Err.	Coef.	Std. Err.	P>z	
AIDs DD *	-0.9822487	0.8625566	-0.6487632	33.72073	-0.3334855	33.73176	0.992	1.66E-07
AIDs+BCID DD *	-1.231096	0.2950153	-1.863535	74.20472	0.6324387	74.20579	0.993	6.38E-10
CT DD *	-1.331235	0.5583234	-2.161197	161.6602	0.8299629	161.6613	0.996	7.72E-07

Publication Bias

The funnel plot employs effect size as the horizontal axis and the reciprocal of the standard error of the effect size as the vertical axis, with the axes labeled in their respective natural scales. Each point in the plot represents an included study. Larger sample sizes lead to more reliable results and smaller variance, resulting in smaller standard errors and a concentration of points in the narrow upper region of the funnel plot. Conversely, smaller sample sizes result in larger variance, greater fluctuation, larger standard errors, and the corresponding points dispersed in the wider lower region of the funnel plot, ultimately forming an inverted funnel shape. The funnel plot allows for direct observation of whether effect size estimates from original studies are associated with their sample sizes. When publication bias exists, the funnel plot exhibits asymmetry, indicating a skewed distribution.

**Figure 18:**
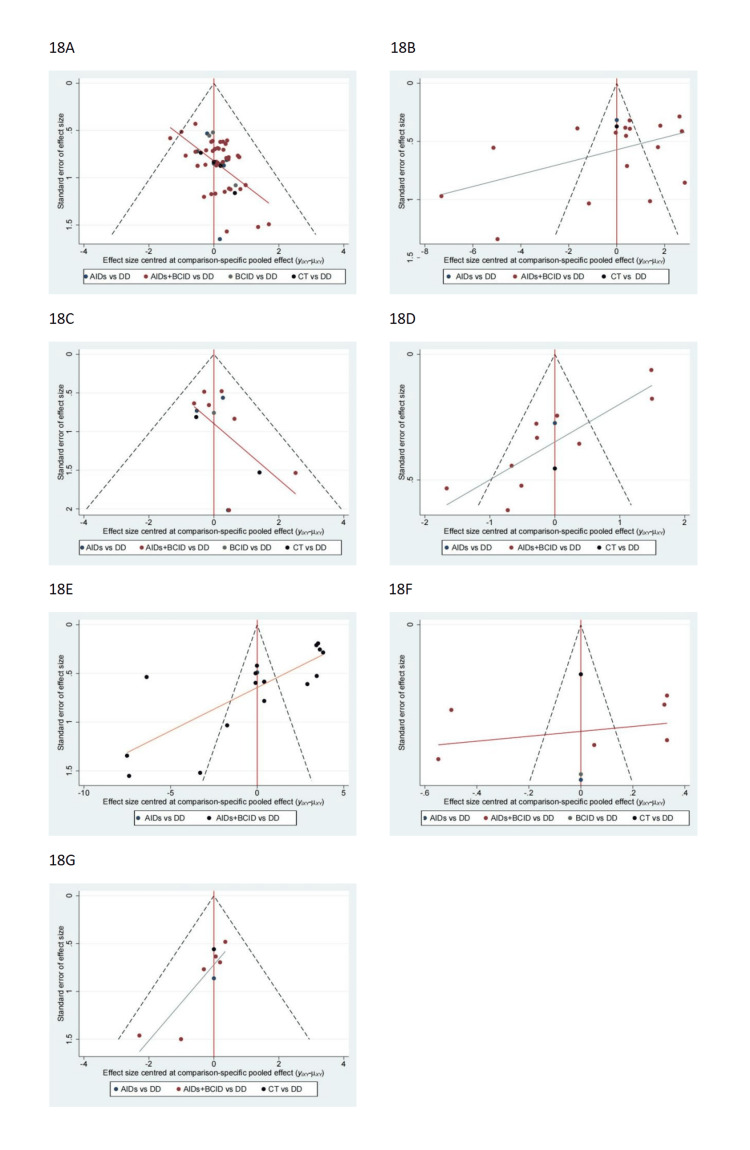
Funnel plot. (A). Overall effective rate of medication. (B) Time to relief or disappearance of the rash. (C) Incidence of adverse reactions. (D) Time to relief or disappearance of abdominal pain. (E) Time to relief or disappearance of arthritic Note: DD: Dihuang Decoction; AIDs: Anti-Inflammatory Drugs; BCID: Blood Circulation Improvement Drug; CT: Conventional Therapy.

Analysis indicates potential publication bias in outcomes related to overall drug efficacy, time to rash relief/disappearance, incidence of adverse reactions, time to abdominal pain relief/disappearance, and time to relief/disappearance of joint swelling and pain. This observation is based on asymmetrical distributions in the funnel plots of these five outcome measures (Figure 18). Such bias could be attributed to the inclusion of numerous studies with small sample sizes. These plots visually represent the potential bias in the respective outcome measures, illustrating a lack of symmetry indicative of publication bias. It is important to note that for IgA levels and the six-month recurrence rate, fewer than 10 studies were included. Therefore, no funnel plots were constructed for these categories.

Discussion

This NMA offers a comprehensive comparison of the efficacy and safety of DD, AIDs, BCIDs, and conventional therapy in managing HSP. The existing body of research in this field is notably deficient, and this study serves to bridge this crucial gap by ranking these treatment methods across various parameters, such as overall drug efficacy, adverse reaction rates, and the time to relief/disappearance of rash, abdominal pain, joint swelling, and pain. It also assesses IgA levels and six-month recurrence rates. Analyzing data from 63 randomized controlled trials, the NMA concludes that DD surpasses other interventions in terms of improving overall medication efficacy and reducing the duration for the relief of rash, abdominal pain, joint swelling, and pain, and lowering adverse reaction rates, IgA levels, and recurrence rates within six months.

The DD is a TCM. Pharmacodynamic studies have shown that dihuang soup has antipyretic, anti-inflammatory, antiallergic, antimetabolic, hepatoprotective, microcirculatory, and immune-enhancing, and reduces vascular endothelial cell adhesion molecules in animals [80]. Laboratory studies have shown that dihuang soup can effectively inhibit platelet endothelial cell adhesion molecule 1, vascular cell adhesion molecule 1, intercellular adhesion molecule 1, and inducible nitric oxide synthase. Nitric oxide synthase secretion, optimizing blood circulation and improving immune function [30]. Xuguang et al. demonstrated that in patients with HSP, pre-treatment sulfate levels were significantly elevated (P<0.05) but reduced after administration of DD (P<0.05). Sulfate maintains cell permeability and regulates intracellular environmental stability. Elevated urinary sulfate concentrations in HSP patients were observed to decrease following treatment, indicating that DD regulates sulfate metabolism in HSP patients [81]. Network pharmacological studies have revealed that the positive regulation of nitric oxide biosynthesis, extracellular space, inflammatory response, oxidative stress, cellular response to tumor factors, peroxidase activity, leukocyte adhesion, and information play crucial roles in the treatment of HSP with DD. This suggests that the core targets interact and influence inflammatory response, oxidative stress, and signaling, thereby affecting cellular proliferation, apoptosis, metabolism, and intercellular adhesion. The primary targets of DD in HSP treatment involve the NF-κB pathway, which is involved in vascular inflammatory and immune responses in HSP patients. The activation level of this pathway is disease-related, and DD can alleviate the inflammatory response by inhibiting the NF-κB signaling pathway [14]. DD mainly alleviates the symptoms of HSP through its antipyretic, anti-inflammatory, anti-allergic reaction, and microcirculation correction effects [56].

Despite its valuable contributions, this NMA has limitations. The literature included is exclusively in Chinese, which may affect the generalizability of the findings due to variations in study quality. Most studies provide insufficient details on randomization methods, allocation concealment, and blinding procedures, raising concerns about potential bias. Additionally, the limited number of studies for certain outcomes might introduce bias in these specific results. Variability in baseline data across studies, such as patient age, disease duration, and intervention course, may impact the reliability of the NMA outcomes. Furthermore, the lack of direct comparisons between interventions in the NMA's network, due to the absence of a closed loop, could diminish the accuracy of the findings.

## Conclusions

In conclusion, while this study supports the efficacy of DD in treating HSP. These research findings indicate that DD could serve as an optimal treatment option for HSP, offering potential guidance for future clinical practice and research. This enables the selection of the most suitable preventive treatment for HSP based on individual patient needs. It also underscores the need for more rigorous research. Future studies should focus on high-quality, large-scale, double-blind randomized controlled trials to further validate and expand upon these NMA results. In addition, future research should delve deeper into the mechanism of action of DD to comprehensively understand how it functions in therapy. Exploring its long-term clinical effects is crucial for assessing its durability and stability throughout treatment. Comparative effectiveness studies with standard therapies are necessary to ensure the rationality and advantages of DD as a treatment option. Furthermore, in-depth investigations into its safety profile are essential to ensure that patients undergoing DD treatment do not experience adverse reactions or complications.
